# H3K27me3‐Mediated Epigenetic Silencing of *FgHMG1* Enables Fungal Host Immune Evasion

**DOI:** 10.1111/pbi.70530

**Published:** 2026-01-06

**Authors:** Xiaozhen Zhao, Bingqin Yuan, Peixue Ma, Yajie Cai, Yan Huang, Yaxuan Wang, Zhen Cheng, Yuan Chen, Minghong Zheng, Ran Zhang, Jinmei Wu, Xieyu Li, Mohan Wang, Huijun Wu, Chengqi Zhang, Xuewen Gao, Li Chen, Qin Gu

**Affiliations:** ^1^ Anhui Province Key Laboratory of Integrated Pest Management on Crops, School of Plant Protection Anhui Agricultural University Hefei China; ^2^ State Key Laboratory of Agricultural and Forestry Biosecurity College of Plant Protection, Nanjing Agricultural University Nanjing China

**Keywords:** epigenetic regulation, *Fusarium graminearum*, H3K27me3 modification, immune evasion, PAMP, plant immunity

## Abstract

Histone H3 lysine 27 trimethylation (H3K27me3) is essential for fungal pathogenicity, yet its contribution to pathogen–host interactions remains incompletely understood. Here, we profiled H3K27me3 dynamics in *Fusarium graminearum* during infection and identified 132 H3K27me3‐marked genes (*FgHMGs*). Among these, *FgHMG1* encodes a secreted glycoside hydrolase family 11 (GH11) protein that functions as a pathogen‐associated molecular pattern (PAMP), triggering PAMP‐triggered immunity (PTI) in *Nicotiana benthamiana* through the receptor kinases BAK1 and SOBIR1, independently of its enzymatic activity. FgHMG1 also induces reactive oxygen species (ROS) accumulation and upregulation of defence‐related genes in wheat plants. Remarkably, FgHMG1 expression is repressed during host invasion by the histone methyltransferase FgKMT6, a homologue of Enhancer of zeste (E(z)) from Drosophila, via H3K27me3 deposition, enabling immune evasion. Loss of FgKMT6 abolishes H3K27me3 enrichment, derepresses FgHMG1, and enhances host immunity, effects largely rescued in *ΔFgKMT6–FgHMG1* double mutants. Notably, foliar application of recombinant FgHMG1 protein reduced Fusarium head blight severity in wheat by 35%–50% in 2‐year field trials. These findings reveal that fungal pathogens exploit H3K27me3‐mediated silencing of immunogenic PAMP genes to evade host recognition and highlight FgHMG1 as a promising candidate for crop protection.

## 1 Introduction

Plants have evolved sophisticated defence strategies to combat pathogenic fungi with diverse lifestyles (Ngou et al. 2022). Plant immunity is initiated when cell surface pattern‐recognition receptors (PRRs) recognise pathogen‐associated molecular patterns (PAMPs), triggering PAMP‐triggered immunity (PTI) as the initial defence line. PTI incorporates a multitude of signalling components that are associated with the production of reactive oxygen species (ROS), Ca^2+^ bursts and the transcription activation of downstream defence‐related genes, thus restricting pathogen entry (Ngou et al. 2021; Yu et al. 2017; Boutrot and Zipfel 2017). However, this recognition imposes strong selective pressure on microbes, driving the evolution of counterstrategies to evade immune detection. Recent research has indicated that pathogens employ various mechanisms to avoid immune detection, such as degrading, modifying, sequestering or suppressing the release of PAMPs (Wang et al. 2022). For example, *Pseudomonas syringae* secretes alkaline protease to degrade flagellin (Pel et al. 2014), or modifies its glycosylation to reduce flg22 release (Buscaill et al. 2019). Fungal pathogens similarly interfere with chitin‐triggered immunity (Jashni et al. 2015): *Cladosporium fulvum* secretes the chitin‐binding effector Ecp6 (de Jonge et al. 2010), *Magnaporthe oryzae* employs Slp1 and *Rhizoctonia solani* produces RsLysM to block chitin perception (Mentlak et al. 2012; Dölfors et al. 2019). In addition, some pathogens regulate PAMP gene expression directly, as seen in *P. syringae* and *F. graminearum* (Bao et al. 2020; Zhao et al. 2024). Yet, the molecular mechanisms underlying such transcriptional regulation of PAMPs remain poorly understood.

Epigenetic modifications provide a dynamic means of controlling gene expression in response to developmental and environmental cues (Zhang and Cao 2019; Bieluszewski et al. 2021; Mushtaq et al. 2021). Among these, histone H3 lysine 27 trimethylation (H3K27me3), deposited by Polycomb repressive complex 2 (PRC2), is a hallmark of transcriptional silencing across eukaryotes (Margueron and Reinberg 2011). The methyltransferase KMT6 (Ezh2) is a core component of PRC2 (Bieluszewski et al. 2021; Wiles and Selker 2017). In fungi, H3K27me3 has emerged as a key regulator of pathogenicity. In *F. graminearum*, KMT6 governs development and controls secondary metabolite gene clusters, including those encoding mycotoxins (Connolly et al. 2013). In *F. fujikuroi*, reduction of H3K27me3 activates numerous metabolite biosynthetic clusters (Studt et al. 2016; Janevska and Tudzynski 2018), while in *M. oryzae* , KMT6‐mediated H3K27me3 is required for growth and virulence (Zhang, Huang, and Cook 2021). Genome‐wide studies show that effectors, enzymes and stress‐response genes are frequently marked by H3K27me3, linking this modification to fungal infection processes (Dong and Ma 2021). In *Zymoseptoria tritici*, effector genes such as *AvrStb6* and *Avr3d1* are repressed by H3K27me3 (Meile et al. 2020), and in *Phytophthora sojae*, silencing of *Avr1b* correlates with H3K27me3 accumulation (Wang et al. 2020). Despite these associations, how pathogens exploit H3K27me3‐mediated silencing to regulate PAMPs and effectors remains largely unknown.

*Fusarium graminearum*, the causal agent of Fusarium head blight (FHB), is a major global threat to wheat production. Climate change and agricultural practices have exacerbated the frequency and severity of FHB outbreaks (Chen et al. 2019; Khan et al. 2020). Previous studies have identified diverse regulators of *F. graminearum* pathogenicity, including transcription factors, kinases, phosphatases, ABC transporters and GPCRs (Jiang et al. 2020, 2019; Gu et al. 2022; Xu et al. 2022; Wang et al. 2019). Yet, how the fungus actively circumvents host immunity during infection is less well understood. In this study, we profiled H3K27me3 dynamics during *F. graminearum* infection and identified 132 H3K27me3‐marked genes (*FgHMGs*). Among them, *FgHMG1* encodes a PAMP recognised by the plant receptor kinases BAK1 and SOBIR1 to activate PTI. We show that FgKMT6‐mediated H3K27me3 represses FgHMG1 expression during infection, enabling immune evasion. Disruption of FgKMT6 abolishes H3K27me3 deposition, derepresses *FgHMG1* and enhances host immunity. Together, our findings demonstrate that FgKMT6‐mediated epigenetic silencing of an immunogenic PAMP represents a critical virulence strategy of *F. graminearum*, providing new insights into host–pathogen interactions and potential avenues for FHB control.

## 2 Results

### 2.1 FgHMG1 With Increased H3K27me3 Modification Triggers Cell Death in *Nicotiana Benthamiana*

**FIGURE 1:**
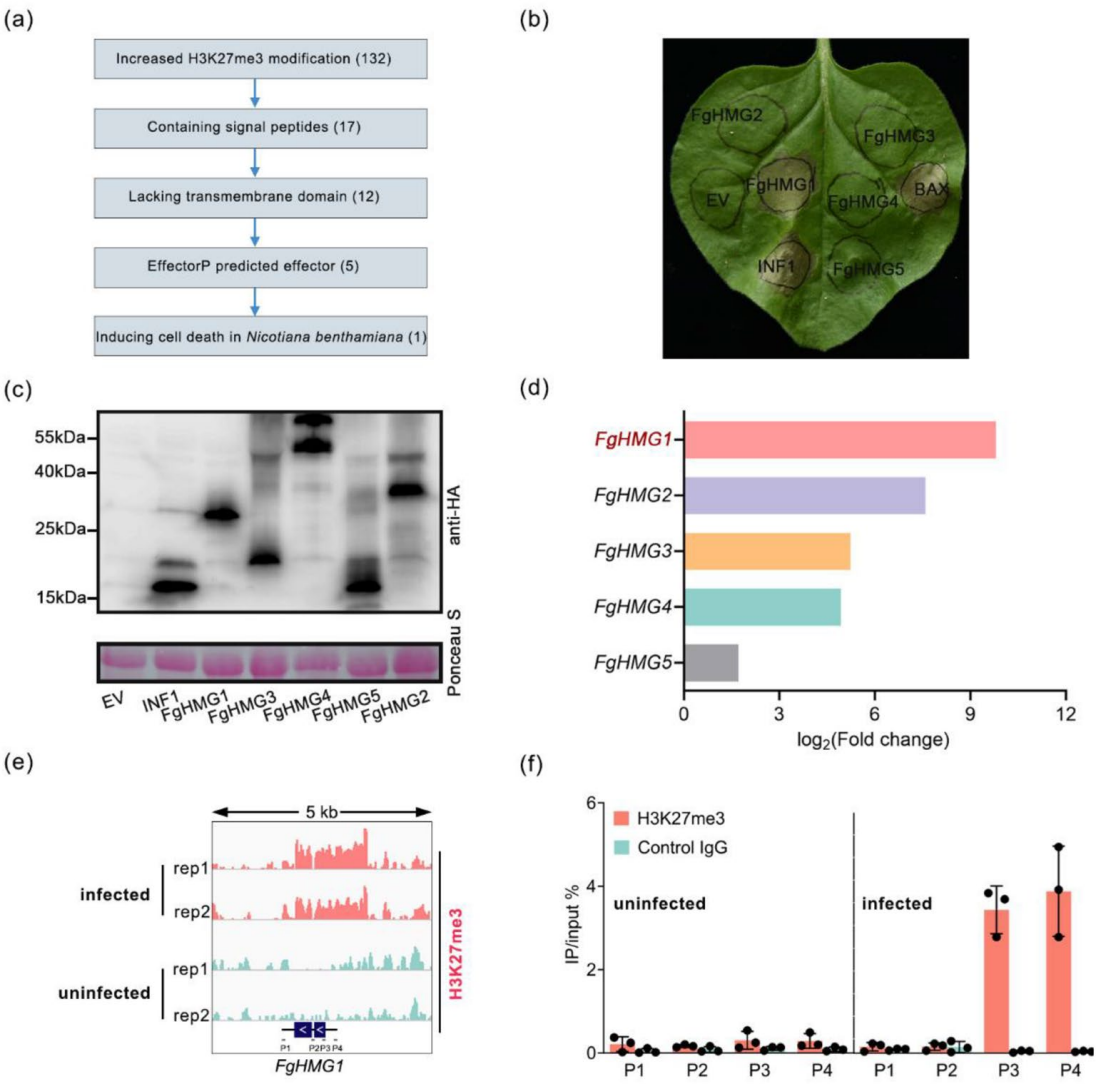
FgHMG1 exhibits the highest increase in H3K27me3 and triggers plant cell death. (a) Screening and identification of putative elicitors based on dynamic profiling of H3K27me3 modifications during *F. graminearum* infection. The number of elicitors identified at each screening step is indicated. (b) Representative *N. benthamiana* leaves exhibiting cell death symptoms at 5 days post‐inoculation (dpi) with *Agrobacterium* strains carrying indicated genes. (c) Immunoblot analysis of transient protein expression in *N. benthamiana* using an anti‐HA antibody (upper panel), with Ponceau S staining shown as the loading control (lower panel). (d) Enrichment levels of H3K27me3 modifications at five candidate elicitor genes under uninfected and *F. graminearum*‐infected conditions. The functional gene *FgHMG1* is labelled in red. (e) Integrative Genomics Viewer of H3K27me3 distribution around *FgHMG1* locus (5 kb around *FgHMG1*) under uninfected and *F. graminearum*‐infected conditions. ChIP‐seq signals were normalised by input using BPM (bins per million mapped reads). The open reading frames of the *FgHMG1* gene are indicated by blue‐shaded regions. To assess the deposition of H3K27me3 across the *FgHMG1* gene, four primer pairs (designated as P1, P2, P3 and P4) were employed. (f) ChIP‐qPCR assays revealing the relative enrichment of H3K27me3 modifications around the *FgHMG1* locus. Relative accumulation levels were represented by percentage of input. Values are the means ± standard deviation (*n* = 3, biologically independent experiments).

To identify effectors regulated by H3K27me3, we built on our previous ChIP‐seq profiling of *F. graminearum* under in vitro (0 h post‐inoculation (hpi), minimal medium) and in planta (48 hpi, wheat heads) conditions (Zhao et al. 2024). Differential binding analysis with DiffBind revealed 132 genes with significantly increased H3K27me3 enrichment during infection (adjusted *p* < 0.05, log_2_[fold change] ≥ 1.5). We next applied a sequential bioinformatic pipeline to prioritise putative effectors among these candidates. SignalP 6.0 screening identified 17 genes encoding secreted proteins with predicted signal peptides. TMHMM v2.0 analysis eliminated proteins with transmembrane domains, leaving 12 candidates. EffectorP 3.0 prediction further refined this set to five high‐confidence effector genes (Figure 1a).

To assess their function, we transiently expressed these five candidates in *N. benthamiana* using PVX‐based vectors under the CaMV 35S promoter. At 5 days post‐infiltration (dpi), only *FgHMG1* (*F. graminearum* H3K27me3‐marked gene 1) triggered visible cell death, whereas the other candidates showed no necrotic response (Figure 1b,c). Notably, FgHMG1 exhibited the strongest increase in H3K27me3 enrichment during infection (Figure 1d).

To validate the histone modification dynamics, we performed ChIP‐qPCR using four primer pairs spanning the *FgHMG1* locus (Figure 1e). Consistent with the ChIP‐seq results, we detected a pronounced accumulation of H3K27me3 signals across both promoter and gene body regions under in planta conditions (Figure 1f). Together, these findings identify FgHMG1 as an H3K27me3‐regulated effector that induces programmed cell death in *N. benthamiana*.

### 2.2 FgHMG1 Triggers Immune Responses

**FIGURE 2:**
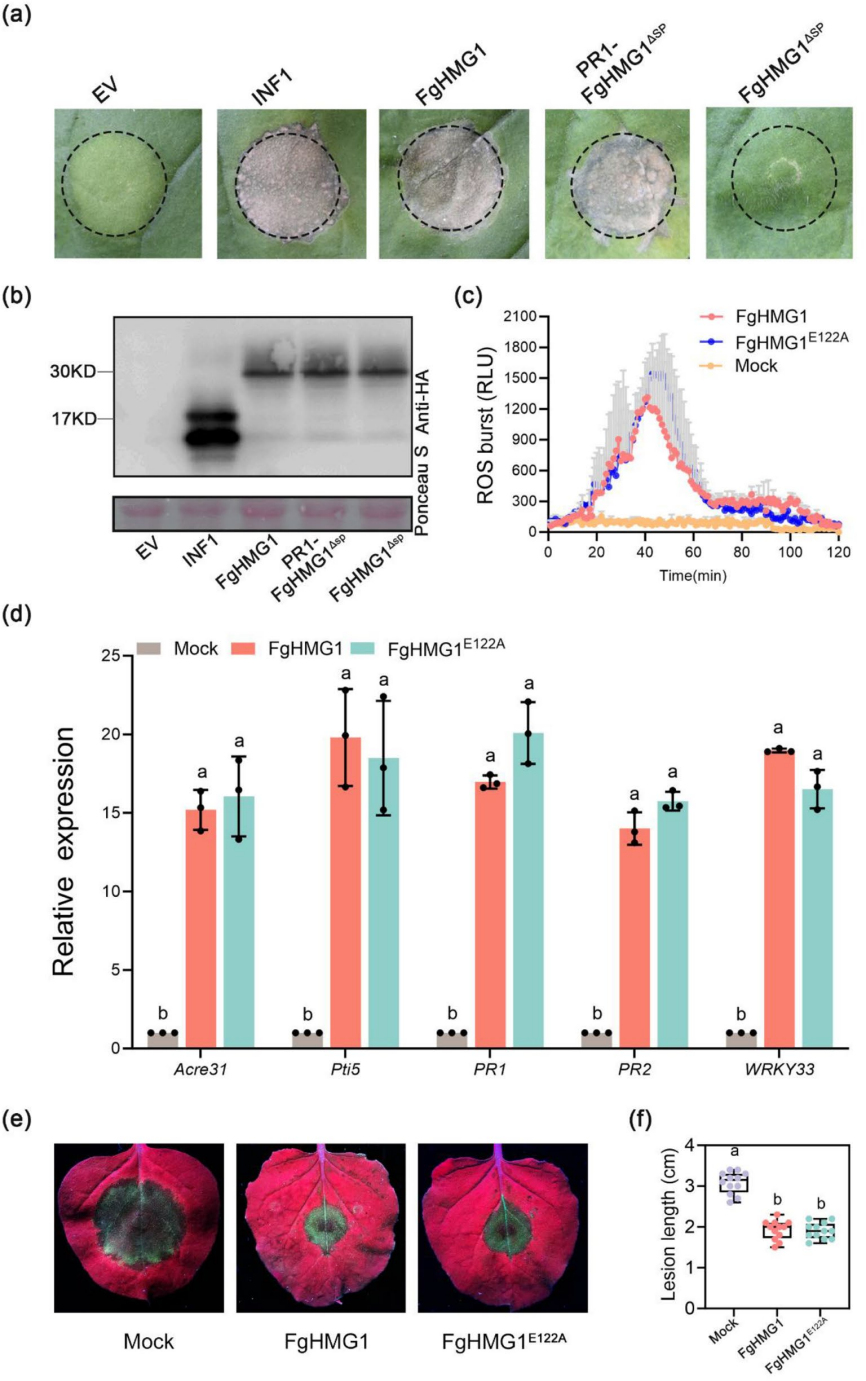
FgHMG1 triggers immune responses in *N. benthamiana* independent of enzymatic activity. (a) Representative *N. benthamiana* leaves exhibiting cell death symptoms at 5 days post‐inoculation (dpi) with *Agrobacterium* strains carrying indicated genes. (b) Immunoblot analysis of transient protein expression in *N. benthamiana* using an anti‐HA antibody (upper panel), with Ponceau S staining shown as the loading control (lower panel). (c) Quantification of ROS accumulation by purified recombinant proteins in *N. benthamiana* leaves. The values are the means ± standard deviation (*n* = 3). (d) Relative expression of defence‐related genes in *N. benthamiana* after infiltrated with 1 μM of indicated recombinant proteins was assessed by RT‐qPCR. *EF‐1α* was used as an internal control. (e, f) Assessment of disease resistance in *N. benthamiana* leaves following transient expression of the FgHMG1. Leaves were inoculated with *P. capsici* 24 h post‐expression. Lesions were examined under UV illumination at 48 h post‐inoculation (e). Quantification of lesion areas was performed using data obtained from 12 technical replicates (f). Box plots display median, interquartile range, min and max. Significant differences (*p* < 0.05, one‐way ANOVA) are indicated by distinct letters.

To determine the role of FgHMG1 in plant immunity, recombinant FgHMG1 protein was purified and infiltrated into *N. benthamiana* mesophyll at concentrations ranging from 0.1 to 8 μM. FgHMG1 induced visible cell death at 4 days post‐infiltration in a dose‐dependent manner (Figure S1). To test whether the apoplastic localisation of FgHMG1 was required to trigger cell death, we generated FgHMG1^ΔSP^ (signal peptide deleted) and PR1‐FgHMG1^ΔSP^ (with the PR1 signal peptide). Transient expression assays showed that FgHMG1^ΔSP^ failed to induce cell death, whereas PR1‐FgHMG1^ΔSP^ restored this activity (Figure 2a). Immunoblotting confirmed proper expression of both constructs at the expected sizes (Figure 2b), indicating that extracellular targeting is essential for FgHMG1‐mediated cell death.

We next examined whether FgHMG1 functions as a PAMP to activate defence responses. Treatment of *N. benthamiana* leaf discs with purified FgHMG1 protein triggered a robust ROS burst (Figure 2c). RT‐qPCR analysis further revealed significant upregulation of defence marker genes, including *Acre31*, *Pti5*, *PR1*, *PR2* and *WRKY33* (Figure 2d).

To test whether FgHMG1 enhances plant defence, we pretreated *N. benthamiana* leaves with FgHMG1 or empty vector (EV) control before inoculation with *Phytophthora capsici*. FgHMG1 pretreatment conferred enhanced resistance, as evidenced by smaller lesion sizes compared with EV controls (Figure 2e,f). Collectively, these results demonstrate that FgHMG1 acts in the apoplast to trigger canonical immune response, including ROS production, defence gene activation and enhanced disease resistance, establishing it as a functional PAMP.

### 2.3 FgHMG1 Triggers Immune Responses in *N. Benthamiana* Independent of Enzymatic Activity

Sequence analysis of FgHMG1 revealed a typical *N*‐terminal signal peptide, a glycoside hydrolase family 11 (GH11) domain, and two predicted catalytic residues, Glu‐122 and Glu‐214 (Figure S2a). To assess their function, we generated two site‐directed mutants, FgHMG1^E122A^ and FgHMG1^E214A^, by substituting the glutamic acid residues with alanine. Recombinant wild‐type and mutant proteins were expressed and purified (Figure S2b). Enzymatic assays confirmed that wild‐type FgHMG1 exhibited xylanase activity in vitro, whereas both mutants lacked detectable activity (Figure S2c). We then investigated whether the ability of FgHMG1 to induce cell death in *N. benthamiana* depends on its xylanase activity. Transient expression of the catalytically inactive mutant FgHMG1^E122A^ in *N. benthamiana* elicited cell death comparable to wild‐type protein (Figure S3a). Western blot analysis confirmed proper accumulation of the mutant protein (Figure S3b). These results indicate that the capacity of FgHMG1 to cause cell death in *N. benthamiana* is independent of its xylanase activity.

Consistently, treatment with FgHMG1^E122A^ also triggered a ROS burst and upregulated defence‐related genes to levels similar to those induced by wild‐type FgHMG1 (Figure 2c,d). Moreover, pretreatment with FgHMG1^E122A^ conferred resistance against *P. capsici*, significantly reducing lesion size to the same extent as wild‐type protein (Figure 2e,f). Together, these results demonstrate that FgHMG1‐mediated immune activation in *N. benthamiana* is independent of its xylanase activity.

### 2.4 BAK1 and SOBIR1 Are Required for FgHMG1‐Triggered Immunity

**FIGURE 3:**
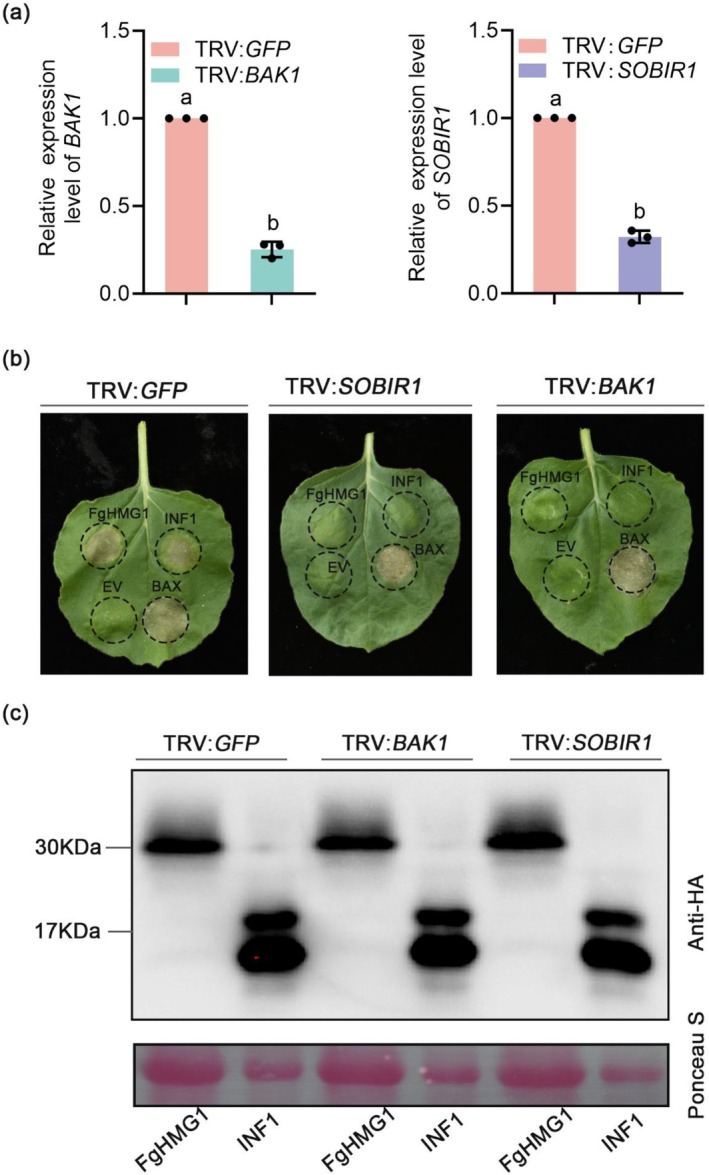
BAK1 and SOBIR1 are required for FgHMG1‐triggered cell death in *N. benthamiana*. (a) Transcript levels of *BAK1* and *SOBIR1* in VIGS‐treated plants, determined by RT‐qPCR using *EF‐1α* as the internal control. Data represent means ± standard deviation (*n* = 3 biological replicates). Different letters indicate statistically significant differences (Student's *t*‐test, *p* < 0.05). (b) Phenotypes of *N. benthamiana* leaves subjected to VIGS with TRV:*BAK1*, TRV:*SOBIR1*, or TRV:*GFP* (control). After 3 weeks, FgHMG1, INF1, EV (empty vector), or BAX were transiently expressed; representative leaves were photographed at 5 days post‐infiltration. (c) Immunoblot detection of FgHMG1 and INF1‐HA expression in silenced leaves. Proteins were detected using an anti‐HA antibody (upper panel). Ponceau S staining of the membrane is shown as the loading control (lower panel).

**FIGURE 4:**
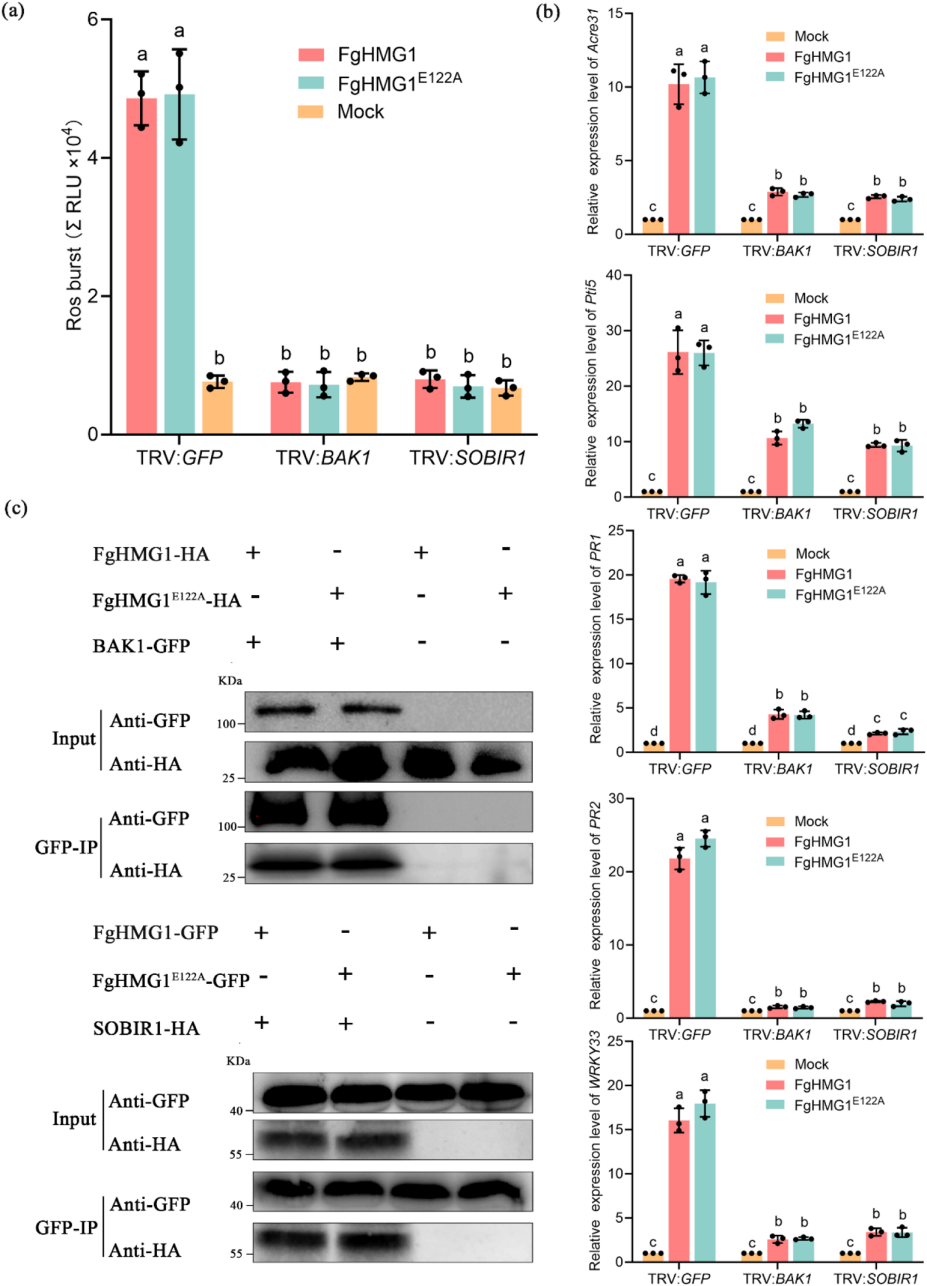
BAK1 and SOBIR1 are required for FgHMG1‐triggered immune responses. (a) The ROS burst triggered by the indicated proteins was measured in *BAK1*‐ and *SOBIR1‐silenced N. benthamiana* leaves. The total relative luminescence units (RLU) were monitored over a 120 min. (b) Relative expression of defence‐related genes in *BAK1*‐ and *SOBIR1‐silenced N. benthamiana* leaves was assessed via RT‐qPCR, with the *EF‐1α* gene serving as the internal reference. In (a, b), the data are presented as means ± standard deviation (*n* = 3), different letters indicate statistically significant differences (*p* < 0.05, one‐way ANOVA). (c) GFP‐ or HA‐tagged FgHMG1 and FgHMG1^E122A^ were co‐expressed with HA‐ or GFP‐tagged BAK1 and SOBIR1 in *N. benthamiana* leaves. The interaction was determined by western blot using anti‐GFP and anti‐HA antibodies.

BAK1 and SOBIR1 act as coreceptors in PRR‐mediated PAMP recognition (Liebrand et al. 2013; Monaghan and Zipfel 2012). To determine their role in FgHMG1 signalling, we silenced *N. benthamiana BAK1* and *SOBIR1* using TRV‐based virus‐induced gene silencing (VIGS), which reduced transcript levels by approximately 68% compared with TRV:*GFP* controls (Figure 3a). In these plants, FgHMG1 failed to trigger cell death, whereas robust cell death occurred in control plants (Figure 3b). This phenotype mirrored the response to INF1, a canonical oomycete PAMP requiring BAK1 and SOBIR1, while BAX induced cell death in all plants irrespective of silencing (Figure 3b). Immunoblotting confirmed expression of INF1 and FgHMG1 at expected sizes (Figure 3c). Consistently, both the ROS burst and defence gene activation normally induced by FgHMG1 were markedly reduced in BAK1‐ and SOBIR1‐silenced plants (Figure 4a,b). The catalytically inactive mutant FgHMG1^E122A^ produced the same pattern, with immune responses abolished in silenced plants (Figure 4a,b).

Next, we performed co‐immunoprecipitation assays and found that both FgHMG1 and FgHMG1^E122A^ interacted with BAK1 and SOBIR1 (Figure 4c). Together, these data demonstrate that FgHMG1 functions as a PAMP of *F. graminearum*, recognised by the plant PRRs BAK1 and SOBIR1 to activate downstream immunity.

### 2.5 FgHMG1 Activates Plant Immunity in Wheat

**FIGURE 5:**
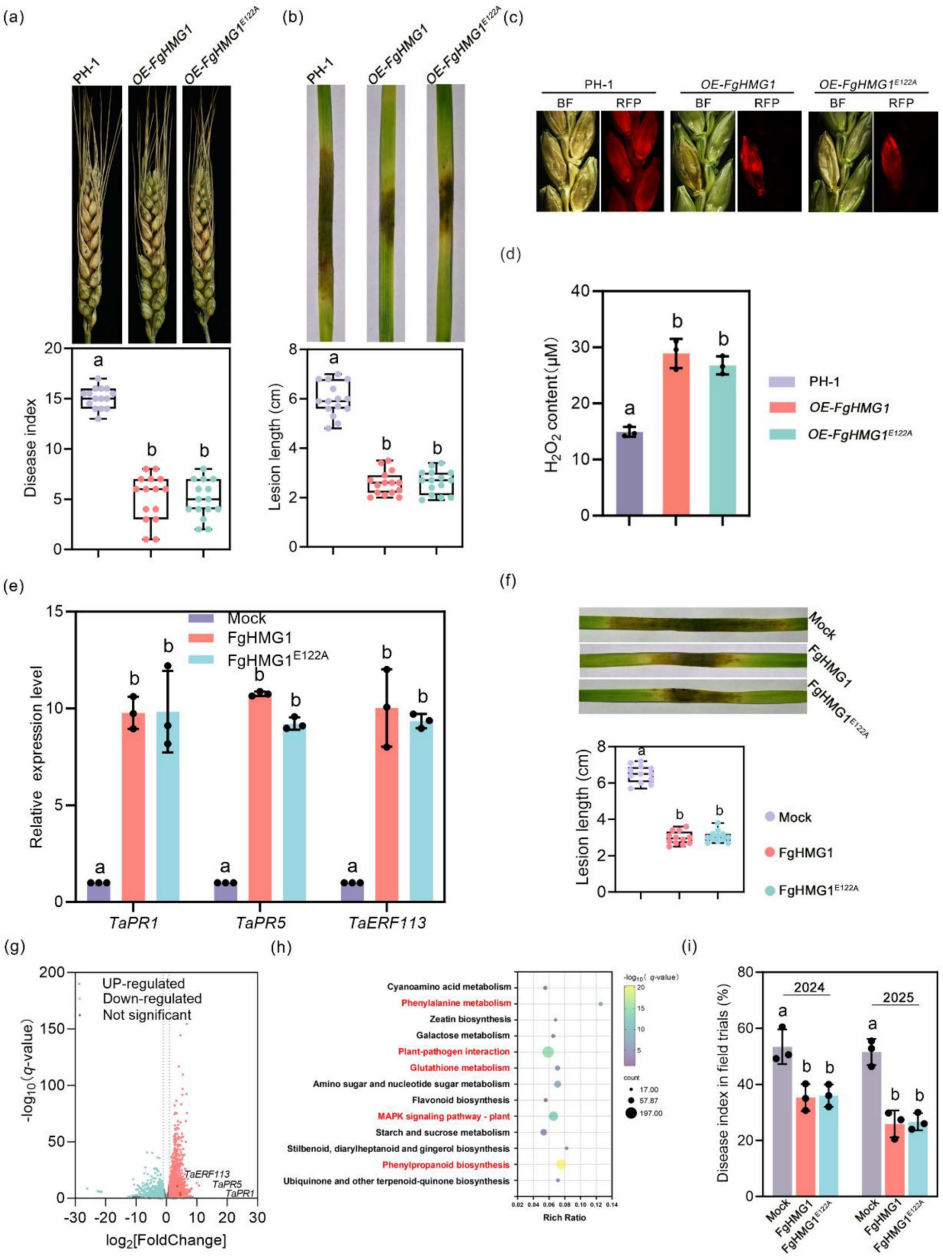
FgHMG1 activates plant immunity in wheat. (a) Pathogenicity assessment on wheat heads inoculated with wild‐type PH‐1 or overexpression strains. Representative pictures were taken at 14 days post‐inoculation (dpi), and the disease index was measured. (b) Pathogenicity on wheat seedling leaves infected with PH‐1 or overexpression strains. Representative photographs were taken at 5 dpi, and lesion lengths were measured. (c) Cross sections of inoculated and adjacent wheat spikelets. Spikelets were inoculated with the indicated strains containing FgACTIN‐RFP. Representative photographs were taken at 7 dpi. BF, bright field; RFP, red fluorescent protein. (d) Quantification of ROS (H_2_O_2_) levels in wheat seedling leaves 48 h post‐inoculation with PH‐1 or overexpression strains. (e) Relative expression levels of defence‐related genes in wheat leaves treated with 1 μM recombinant proteins were assessed by RT‐qPCR. *TaGAPDH* was used as an internal control. (f) Disease resistance in wheat seedling leaves triggered by pretreatment with 1 μM indicated proteins, followed by PH‐1 inoculation. 1 μM of GFP protein was used for the control. Symptoms were shown at 5 dpi and lesion lengths were measured. (g) Transcriptome analysis of DEGs (differentially expressed genes) in FgHMG1‐treated wheat seedling leaves (log_2_[fold change] ≥ 1 or ≤ −1, *q*‐value < 0.05). Key defence genes (*TaPR1*, *TaPR5* and *TaERF113*) are highlighted. (h) KEGG enrichment analysis of upregulated genes. (i) Fusarium head blight disease index following pretreatment with 1 μM FgHMG1 or FgHMG1^E122A^ in 2024 and 2025 field trials. 1 μM of GFP protein was used for the untreated control (Mock). In (a, b, f) data represent mean ± standard deviation (SD) (*n* = 15 for a and b; *n* = 12 for f). Box plots display median, interquartile range, min, and max. Significant differences (*p* < 0.05, one‐way ANOVA) are indicated by distinct letters. In (d, e, i) data presented are the means ± SD (*n* = 3). Statistical significance (*p* < 0.05) determined by one‐way ANOVA.

To evaluate whether FgHMG1 can activate immunity in wheat, the natural host of *F. graminearum*, we overexpressed FgHMG1 under the *gpda* promoter in PH‐1. The overexpression strain (*OE‐FgHMG1*) displayed > 25‐fold higher transcript levels compared with wild‐type (WT) (Figure S4a), without affecting colony morphology or growth (Figure S4b). However, *OE‐FgHMG1* showed markedly reduced pathogenicity on wheat spikes and seedling leaves (Figure 5a–c). Infected wheat tissues exhibited stronger H_2_O_2_ accumulation relative to WT (Figure 5d). Similarly, exogenous application of FgHMG1 protein suppressed *F. graminearum* pathogenicity and induced expression of wheat defence‐related genes (Figure 5e,f). Moreover, the protection conferred by FgHMG1 was dose‐dependent, with a threshold concentration of at least 300 nM required for a significant reduction in disease development (Figure S5). Collectively, these findings indicated that FgHMG1 contributes to plant resistance in wheat.

To test whether enzymatic activity is required, we generated an overexpression strain of the xylanase‐inactive mutant (*OE‐FgHMG1* ^*E122A*^). This strain also showed attenuated virulence and induced H_2_O_2_ accumulation (Figure 5a–d). Foliar application of FgHMG1^E122A^ protein further enhanced wheat resistance and activated defence marker genes to a similar extent as the wild‐type protein (Figure 5e,f). Thus, FgHMG1‐mediated immunity is independent of xylanase activity.

RNA‐seq analysis revealed that FgHMG1 treatment upregulated genes enriched in defence responses and immune system processes (Figure 5g,h). Importantly, 2‐year field trials in 2024 and 2025 showed that foliar application of either FgHMG1 or FgHMG1^E122A^ conferred 35%–50% control efficacy against Fusarium head blight (FHB) (Figure 5i). Together, these results establish FgHMG1 as a fungal PAMP that activates wheat immunity and an elicitor for FHB control.

We next evaluated the potential of FgHMG1 as a broad‐spectrum protectant. Pretreatment of rice leaves with FgHMG1 significantly reduced disease symptoms caused by the blast fungus *Magnaporthe oryzae* (Figure S6), indicating that the induced immunity is effective even against pathogens from distinct genera. To further explore the basis of this broad‐spectrum activity, we analysed homologues from 12 major plant‐pathogenic fungi and found that FgHMG1 is highly conserved (Figure S7), suggesting that its immune‐inducing function may be widely retained across fungal species. Because protein stability is essential for practical application, we further assessed the thermal stability of FgHMG1 and found that it retained immunogenic activity after incubation across a range of temperatures (Figure S8). Together, the high sequence conservation and strong thermal stability underscore the potential of FgHMG1 as a broad‐spectrum disease protectant.

### 2.6 H3K27me3‐Mediated Repression of FgHMG1 Enables Immune Evasion

**FIGURE 6:**
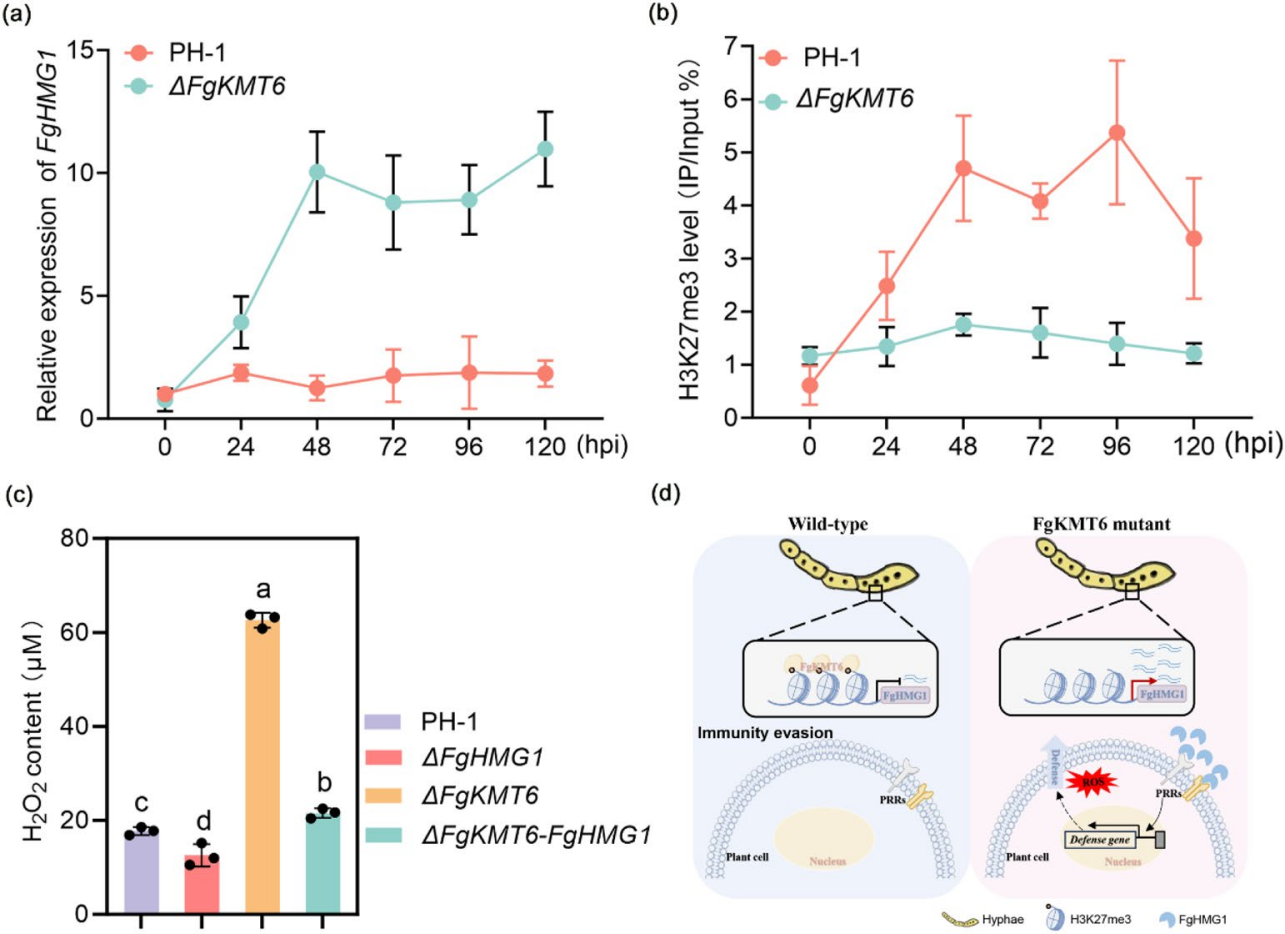
H3K27me3‐mediated repression of F*gHMG1* enables immune evasion. (a) Relative expression of *FgHMG1* assessed by RT‐qPCR in wild‐type PH‐1 and the *ΔFgKMT6* mutant post‐inoculation (0–120 h post‐inoculation (hpi)). The data are presented as means ± standard deviation (SD) (*n* = 3). (b) H3K27me3 deposition at *FgHMG1* measured by ChIP‐qPCR in wild‐type PH‐1 and the *ΔFgKMT6* mutant (0–120 hpi). Relative accumulation levels are shown as percentage of input. (c) Quantification of ROS (H_2_O_2_) levels in wheat seedling leaves 48 hpi with PH‐1 or the indicated strains. Data represent mean ± SD from three independent experiments. Different letters denote significant differences (*p* < 0.05, one‐way ANOVA). (d) Model of an H3K27me3‐dependent silencing strategy evolved in *F. graminearum* to evade host immunity and promote infection.

To explore how *F. graminearum* suppresses FgHMG1‐triggered immunity, we examined its transcriptional dynamics during infection. *FgHMG1* expression remained low in wild‐type (WT) PH‐1 (Figure 6a), coinciding with strong H3K27me3 enrichment at its locus (Figure 6b). These data suggest that epigenetic repression via H3K27me3 prevents *FgHMG1* activation during host colonisation.

To test this, we analysed an FgKMT6 deletion mutant lacking the H3K27 methyltransferase. In *ΔFgKMT6*, *FgHMG1* was strongly derepressed (Figure 6a) and H3K27me3 enrichment at its locus was abolished (Figure 6b), confirming that FgKMT6 directly mediates its repression. To further establish the direct mechanistic link, we performed ChIP‐qPCR assays to determine the occupancy of FgKMT6 at the *FgHMG1* locus during infection using the PH‐1::FgKMT6‐GFP strain. The results showed that FgKMT6‐GFP was highly enriched at the *FgHMG1* locus from 24 to 120 hpi (Figure S9), which coincides with H3K27me3 marks establishment. The finding indicates that histone methyltransferases FgKMT6 are recruited to the *FgHMG1* locus to establish the repressive H3K27me3 modification. Functionally, *ΔFgKMT6* infection resulted in elevated H_2_O_2_ accumulation in wheat compared to WT, reflecting enhanced immune activation (Figure 6c). Importantly, this phenotype was abolished in the double mutant *ΔFgKMT6‐FgHMG1* (Figure 6c), demonstrating that *FgHMG1* derepression accounts for the heightened host response. Together, these findings establish that *F. graminearum* employs FgKMT6‐mediated H3K27me3 to silence *FgHMG1*, thereby attenuating its immune elicitation and facilitating successful infection (Figure 6d).

## 3 Discussion

Plants have evolved sophisticated mechanisms to recognise cell wall‐degrading enzymes (CWDEs) as pathogen‐associated molecular patterns (PAMPs) through plasma membrane receptors, thereby activating pattern‐triggered immunity (PTI). For example, the glycosyl hydrolase XEG1 from *P. sojae* is recognised by the leucine‐rich repeat receptor‐like protein (LRR‐RLP) NbRXEG1 in *N. benthamiana*, initiating immune signalling cascades (Wang et al. 2018). Similarly, FoEG1, a cellulase‐active enzyme from *F. oxysporum*, functions as a PAMP that induces cell death, reactive oxygen species (ROS) production and callose deposition (Zhang, Yan, et al. 2021). Other CWDEs, including GH12 xyloglucanases BcXYG1 and BcXyl1 from *Botrytis cinerea* and multiple hydrolases from *Verticillium dahliae*, have also been reported to elicit immune responses (Zhu et al. 2017; Yang, Yang, et al. 2018; Gui et al. 2017, 2018; Yang, Zhang, et al. 2018). Our findings add a GH11 xylanase, FgHMG1, to this repertoire, demonstrating that it functions as a PAMP that activates cell death, ROS accumulation and defence gene expression in *N. benthamiana* independently of its enzymatic activity. This finding expands our understanding of the molecular mechanisms underlying plant‐pathogen interactions and highlights the diverse array of PAMPs that plants can perceive to mount effective immune responses.

The apoplast serves as a key interface for plant‐pathogen interactions. Many effectors require correct secretion to exert immune functions (Doehlemann and Hemetsberger 2013). For instance, RcCDI1‐mediated cell death depends on its signal peptide (Franco‐Orozco et al. 2017), while VmE02 from *Valsa mali* and SsCP1 from *Sclerotinia sclerotiorum* act independently of secretion (Nie et al. 2019; Yang, Tang, et al. 2018). We show that FgHMG1‐triggered cell death also depends on its signal peptide, highlighting the functional diversity of effector deployment strategies in the apoplast.

PAMP perception typically relies on receptor‐like kinases (RLKs) or receptor‐like proteins (RLPs) (Tang et al. 2017), which often recruit co‐receptors such as BAK1 and SOBIR1 to transduce immune signals (Liebrand et al. 2014; van der Burgh et al. 2019). Previous studies established their roles in LeEIX1‐, SlEIX2‐, and AtRLP42‐mediated immunity (Liebrand et al. 2013; Bar et al. 2010; Zhang et al. 2014). In line with these findings, we demonstrate that FgHMG1‐triggered cell death, ROS burst and defence gene activation in *N. benthamiana* require both BAK1 and SOBIR1, and that FgHMG1 physically associates with these co‐receptors. Interestingly, this mechanism contrasts with the GH11 protein VdEIX3 from *V. dahliae*, which induces immunity independently of BAK1 and SOBIR1 (Yin et al. 2021), reflecting the evolutionary diversity of plant immune recognition systems.

The plant‐pathogen arms race continuously drives innovation in recognition and evasion strategies. Pathogens frequently escape immune detection by effector point mutations, as seen in *P. sojae* Avr3c or *Leptosphaeria maculans* AvrLm4‐7 (Huang et al. 2019; Parlange et al. 2009), or by transcriptional suppression of effectors such as PsAvr3a (Shrestha et al. 2016). Our study reveals that *F. graminearum* employs an alternative strategy: epigenetic repression of *FgHMG1* via H3K27me3, mediated by the histone methyltransferase FgKMT6. Loss of FgKMT6 abolishes H3K27me3 deposition, derepresses *FgHMG1* and enhances immune activation in wheat, whereas deleting both FgKMT6 and FgHMG1 restores immune evasion. This highlights H3K27me3 as a critical regulatory layer that pathogens exploit to fine‐tune PAMP expression and circumvent host defences. The complexity of this regulatory layer is further supported by our finding that the phenotype of the *ΔFgKMT6‐FgHMG1* double mutant was only partially restored. Such incomplete complementation indicates that FgKMT6‐mediated H3K27me3 repression targets additional immunogenic factors beyond FgHMG1. Consistent with this, a recent study demonstrated that FgKMT6 also suppresses the PAMP BCG1, thereby promoting fungal invasion and immune evasion (Zhao et al. 2024). These examples underscore the adaptability of pathogens, emphasising the crucial need for further research into their mechanisms of evading plant immunity.

Histone modifications, including H3K27me3, are widely recognised as regulators of stress adaptation and host–microbe interactions in both plants and fungi (Ruthenburg et al. 2007; Hannan Parker et al. 2022; Gómez‐Díaz et al. 2012). In *Neurospora crassa*, H3K27me3 modulates responses to genotoxic stress (Jamieson et al. 2013); in *Epichloë festucae*, it represses alkaloid biosynthesis to maintain host compatibility (Chujo and Scott 2014); and in *P. sojae*, it silences *Avr1b* to evade immunity (Wang et al. 2020). Our results extend this paradigm by showing that H3K27me3 dynamically represses a PAMP gene in *F. graminearum*, establishing chromatin‐level silencing as a virulence strategy.

Consistent with animal systems, FgKMT6, the fungal ortholog of EZH2, is indispensable for H3K27me3 deposition (Connolly et al. 2013). Its deletion leads to derepression of *FgHMG1* and enhanced immune activation, demonstrating its central role in coordinating pathogen epigenomes with infection success. These findings point to epigenetic regulators as potential antifungal targets. However, key questions remain regarding how FgKMT6 is recruited to specific genomic loci and whether additional chromatin regulators cooperate in effector silencing. Addressing these questions could uncover novel vulnerabilities for disease control.

In this study, FgHMG1 confers broad‐spectrum resistance, protecting wheat and rice against pathogens from different genera, such as *F. graminearum* and *Magnaporthe oryzae*. The molecular basis for this wide‐ranging activity likely resides in its high conservation across diverse plant‐pathogenic fungi, which enables its broad recognition by plant immune systems. Furthermore, for practical application, FgHMG1 exhibits relatively high stability, retaining its immunogenic activity under varying temperatures. Together, its strong conservation and stability not only support broad immune recognition but also highlight its promise as a widely applicable plant protectant.

Our field trials showed that exogenous application of FgHMG1 and its enzymatically inactive mutant reduced Fusarium head blight severity in wheat by 35%–50%, highlighting its potential as a protein‐based elicitor for sustainable crop protection. Together, this study uncovers an epigenetically regulated PAMP in *F. graminearum* and establishes a framework linking histone modifications to immune evasion and disease management strategies.

## 4 Materials and Methods

### 4.1 Fungal Strains and Culture Conditions

The *F. graminearum* wild‐type strain PH‐1 (NRRL 31084) served as the parental reference throughout this study. Mycelial growth of PH‐1 and its derivatives was evaluated on potato dextrose agar (PDA, 200 g potato, 20 g glucose, 20 g agar, 1 L water) at 25°C. To generate fresh conidia, mycelial plugs from the periphery of 3‐day‐old colonies were transferred to 100 mL carboxymethyl cellulose (CMC) liquid medium (15 g CMC‐Na, 0.5 g (NH_4_)_2_SO_4_, 1 g NaNO_3_, 1 g KH_2_PO_4_, 0.5 g MgSO_4_·7H_2_O, 1 L water) and incubated at 25°C with shaking (180 rpm) for 4 days. Conidial concentrations were quantified using a haemocytometer.

### 4.2 Pathogenicity Assays

The pathogenicity of each strain on wheat spikelets and seedling leaves was assessed using previously described protocols (Gu et al. 2022). For wheat head infections, conidial suspensions (1 × 10^6^ conidia/mL) were applied via point‐inoculation. Infected spikelets were documented, and representative images captured 14 days post‐inoculation (dpi). For seedling leaf assays, 5‐mm mycelial plugs from 3‐day‐old PDA cultures were inoculated onto leaves. Disease lesion development was quantified and photographed at 5 dpi. All pathogenicity assays were conducted in triplicate to ensure reproducibility.

### 4.3 ChIP‐Seq and ChIP‐qPCR

ChIP experiments were conducted as previously reported (Zhao et al. 2024). Fresh mycelia (5 g) cultivated in Fusarium minimal medium (FMM, 0.5 g KCl, 2 g NaNO_3_, 1 g KH_2_PO_4_, 0.5 g MgSO_4_·7H_2_O, 30 g sucrose, 200 μL trace elements for 1 L) or wheat heads (48 h post‐inoculation) were harvested for nuclei isolation. Chromatin was digested using 15 μL micrococcal nuclease (MNase; NEB M0247S) at 37°C for 4 min, followed by immediate termination with 250 μL 0.5 M EDTA. After centrifugation, 200 μL supernatant was reserved as input DNA and stored at −20°C. Immunoprecipitation was performed overnight at 4°C using anti‐H3K27me3 (Thermo Fisher Scientific, 39 155; 1:500) antibody conjugated to protein A agarose beads (Santa Cruz Biotechnology, sc‐2001). Antibody‐bead complexes were sequentially washed with 1 mL each of low‐salt buffer A (50 mM Tris–HCl, 10 mM EDTA, 50 mM NaCl, pH 7.5), moderate‐salt buffer B (100 mM NaCl), and high‐salt buffer C (150 mM NaCl). DNA was eluted with 400 μL elution buffer (50 mM NaCl, 20 mM Tris–HCl, 1% SDS, 5 mM EDTA, pH 7.5) at 65°C for 20 min. ChIP DNA (H3K27me3) and input controls were purified using phenol/chloroform extraction. Libraries were prepared and sequenced on the Illumina HiSeq 4000 platform (BGI, Shenzhen, China), while the other half was used for ChIP‐qPCR.

For ChIP‐seq analysis, the raw sequencing reads were quality‐checked using SOAPnuke (v. 2.1.7) (Chen et al. 2018) and were aligned to the *F. graminearum* PH‐1 reference genome using Bowtie2 (v. 2.4.5) under default parameters (Langmead and Salzberg 2012). Reads were normalised as BPM (Bins Per Million mapped) reads via Deeptools' bamCompare (v2.4.1) using input DNA controls (Ramírez et al. 2014). Resulting bigwig files were visualised and analysed using the Integrative Genomics Viewer (IGV v2.8.9) (Robinson et al. 2011). Peak calling was performed with MACS2 (v2.1.4) using established parameters (Zhang et al. 2008; You et al. 2017).

For ChIP‐qPCR analysis, the relative enrichment was determined by normalising the immunoprecipitated DNA quantity to the input DNA quantity. PCR primer pairs were designed to amplify 110–160 bp fragments encompassing the target genomic loci (Table S1). The ChIP‐qPCR experiments were performed in triplicate.

### 4.4 Co‐Immunoprecipitation (Co‐IP) Assay

To investigate the interaction of FgHMG1/FgHMG1^E122A^ with BAK1 or SOBIR1, transient co‐expression of these putative interacting proteins was performed in *Nicotiana benthamiana* via *Agrobacterium*‐mediated infiltration. Leaf tissues were flash‐frozen in liquid nitrogen and homogenised, followed by total protein extraction using cell lysis buffer (Beyotime, p0043) supplemented with 0.1% (v/v) protease inhibitor cocktail (P9599; Sigma, St. Louis, MI, USA) for 20 min at 4°C. After centrifugation at 14 000 × g for 15 min, 200 μL of supernatant was boiled for 5 min as input. For Co‐IP, pre‐cleared supernatants were incubated overnight at 4°C with GFP‐Trap A beads (Chromotek, Hauppauge, NY, USA, gta‐20). Bead‐bound immunocomplexes were collected by centrifugation and subjected to three sequential washes with Tris‐buffered saline (TBS). Immunoblotting was performed using anti‐HA (M20003M, Abmart, Shanghai, China, 1:10000 dilution) and anti‐GFP (ab32146, Abcam, Cambridge, UK, 1:10000 dilution) antibodies.

### 4.5 RNA‐Seq Analysis

For transcriptome analysis of wheat plants, the wheat seedling leaves were treated with 1 μM of FgHMG1 protein or GFP control (mock), and samples were collected after 24 h. Total RNA was isolated using TRIZOL reagent (TaKaRa, Dalian, China) according to the manufacturer's instructions, and subsequent library preparation and sequencing were performed on an Illumina HiSeq 4000 platform. RNA‐seq data analysis was performed using the BGI online platform. Raw reads were quality‐filtered with SOAPnuke, and then aligned to the reference genome of wheat *Triticum aestivum* cv. Chinese Spring (IWGSC RefSeq v2.1) using HISAT2. Differential expression analysis was performed using DESeq2 (v.1.4.5) with *q*‐value < 0.05 and log_2_[fold change] ≥ 1 or ≤ −1, and the results were visualised in volcano plots. Finally, the KEGG pathway enrichment analysis of the upregulated genes was performed using the DAVID database integrated with the BGI platform tools. The RNA‐seq data have been deposited in the NCBI BioProject database with accession code GSE304049 (https://www.ncbi.nlm.nih.gov/geo/query/acc.cgi?acc=GSE304049, Enter token azgbmygsfdezpqb into the box).

### 4.6 Quantitative Real‐Time PCR (qRT‐PCR)

Total RNA was extracted using the TRIZOL reagent (TaKaRa Biotechnology Co. Ltd., Dalian, China) following the manufacturer's protocol. First‐strand cDNA was generated using the Primescript first‐strand cDNA synthesis system (TaKaRa, Dalian, China) with oligo(dT) primers in 20 μL reaction volumes. qRT‐PCR reactions were performed in technical triplicates using iTaq SYBR Green Master Mix (Bio‐Rad, Hercules, CA, USA). Amplification parameters were: initial denaturation at 95°C for 30 s, followed by 40 cycles of 95°C for 5 s and 60°C for 15 s. The *FgACTIN* gene served as an endogenous reference for normalisation, and relative expression levels were determined using the $2^{-\Delta \Delta C_{t}}$ method. All primer sequences are listed in Table S1.

### 4.7 Protein Purification and Xylanase Activity Assays

The full‐length open reading frame (ORF) of FgHMG1 was inserted into the pET28a‐GFP expression vector and transfected into competent *E. coli* BL21 (DE3) cells. A 20‐mL preculture of transformed bacteria was grown in LB medium at 37°C with shaking at 180 rpm for 12 h. This preculture was then diluted 1:100 into 200 mL fresh LB medium and incubated under identical conditions until the optical density at 600 nm (OD₆₀₀) reached 0.6. Protein expression was induced by adding 0.5 mM isopropyl β‐D‐1‐thiogalactopyranoside (IPTG), followed by overnight incubation at 16°C with shaking at 180 rpm. Bacterial pellets were harvested via centrifugation (5000 × g, 4°C, 30 min) and resuspended in either Buffer A or phosphate‐buffered saline (PBS). Following sonication‐mediated cell lysis, clarified supernatants were obtained through centrifugation (10 000 × g, 4°C, 20 min) and subjected to affinity purification using an ÄKTA avant 25 system (GE Healthcare). Protein expression profiles were verified by sodium dodecyl sulphate polyacrylamide gel electrophoresis (SDS‐PAGE). Analogous procedures were employed for the expression and purification of FgHMG1^E122A^, FgHMG1^E214A^ recombinant proteins.

The enzyme activity of purified proteins was further determined by the 2,4‐dinitrosalicyclic acid (DNS) assay, as reported in Moscetti et al. (2013).

### 4.8 ROS Accumulation Assays

To assess reactive oxygen species (ROS) accumulation in wheat plants following *F. graminearum* infection, leaf tissues were inoculated with wild‐type PH‐1, overexpressing strains (*OE‐FgHMG1*, *OE‐FgHMG1* ^*E122A*^), *ΔFgHMG1* mutant, *ΔFgKMT6* mutant or *ΔFgKMT6‐FgHMG1* strains for 48 h. For quantitative hydrogen peroxide determination, leaf homogenates (100 mg/mL PBS) were centrifuged at 12 000 × g (15 min, 4°C); 50 μL supernatant was reacted with 100 μL detection reagent from a commercial H_2_O_2_ assay kit (S0038, Beyotime Biotechnology) at 25°C for 25 min. Absorbance at 565 nm was measured and H_2_O_2_ concentrations calculated against a standard curve.

ROS burst was monitored using a luminol/peroxidase detection system. Leaf discs from 5‐week‐old *N. benthamiana* were pre‐incubated overnight in 200 μL sterile water, then transferred to reaction solution containing 17 μg/mL luminol, 10 μg/mL horseradish peroxidase and 1 μM purified recombinant protein. Luminescence was quantified using a GLOMAX96 microplate luminometer (Promega, Madison, WI, USA).

### 4.9 Agroinfiltration and Virus‐Induced Gene Silencing (VIGS) Assay in *N. Benthamiana*

Transient expression assays in *N. benthamiana* were conducted following a previously established protocol (Yu et al. 2019). For FgHMG1‐induced cell death analysis, the FgHMG1 coding sequence was cloned into pGR107 and transformed into *Agrobacterium tumefaciens* strain GV3101. The transformed bacteria were cultured in LB liquid medium at 28°C for 16 h, harvested by centrifugation, and resuspended in infiltration buffer (10 mM MES, 10 mM MgCl_2_ and 150 μM acetosyringone). Bacterial suspensions were standardised to an OD₆₀₀ of 0.4, incubated at room temperature for 2 h, and infiltrated into the abaxial leaf surfaces of 5‐week‐old *N. benthamiana* plants using a needleless syringe. Leaf tissues were collected 48 h post‐infiltration for protein extraction. For VIGS experiments, equal volumes of *Agrobacterium* cultures harbouring TRV2:*GFP* (control), TRV2:*BAK1* or TRV2:*SOBIR1* vectors were mixed with cultures containing the TRV1 vector. The mixed suspensions were infiltrated into *N. benthamiana* seedlings, and silencing efficiency was assessed by RT‐qPCR analysis of target gene expression levels. All assays were performed in triplicate.

### 4.10 Induced Resistance Assays

To determine whether FgHMG1 protein induces resistance in rice against *Magnaporthe oryzae*, 2‐week‐old rice seedlings ( *Oryza sativa* CO39) were sprayed with a solution of purified FgHMG1 protein or, as a control, with GFP protein. Twenty‐four hours after protein treatment, the plants were challenge‐inoculated with *M. oryzae* by spraying with a conidial suspension (5 × 10^4^ spores per ml in 0.2% gelatin). The inoculated plants were maintained in a growth chamber at 25°C and 90% humidity, kept in darkness for the first 24 h and then under a 12/12 h light/dark cycle. Disease severity was evaluated based on lesion development at 5 days post‐inoculation.

### 4.11 Statistical Analysis

The statistical analysis was performed using SPSS software (version 21). Each experiment was conducted in triplicate, and the resulting data were subjected to one‐way analysis of variance (ANOVA) with a significance threshold of *p* < 0.05.

## Author Contributions

Q.G., L.C. and X.Z. designed the experiments. P.M., Y.C., Y.H., Y.W., Z.C., Y.C., M.Z., R.Z., J.W., X.L. and M.W. performed the experiments. X.Z. and B.Y. analysed data. Q.G., L.C. and X.Z. wrote the manuscript. H.W., C.Z. and X.G. supervised the subject.

## Funding

This work was financially supported by the National Key R&D Program of China (2024YFD1401103 and 2022YFD1400102); the National Natural Science Foundation of China (32472535 and 32402305); the Natural Science Foundation for Distinguished Young Scholars of Jiangsu Province (BK20240088); Fundamental Research Funds for the Central Universities (KJJQ2025025); the Talent Research Project of Anhui Agricultural University (rc342502); the Joint Research Program of State Key Laboratory of Agricultural and Forestry Biosecurity (SKLJRP2510); and the Science and Technology Planning Project of Jurong City (ZA32320).

## Conflicts of Interest

The authors declare no conflicts of interest.

## Supporting information


**Figure S1:** FgHMG1 induces cell death in *N. benthamiana*. Purified FgHMG1 protein (8 μM to 0.1 μM) or a GFP‐tagged control protein was infiltrated into *N. benthamiana* leaves. Representative leaf phenotypes photographed at 4 days post‐infiltration (dpi) (upper panel), corresponding leaves stained with trypan blue (lower panel).
**Figure S2:** Xylanase activity of FgHMG1 and its site‐directed mutants. (a) Domain architecture of *F. graminearum* FgHMG1. (b) Expression and purification of GFP‐tagged proteins. (c) In vitro assays for the xylanase activity of indicated recombinant proteins. Xylanase activity of indicated proteins was determined using the 2,4‐dinitrosalicyclic acid (DNS) assay. The data are presented as means ± standard deviation (*n* = 3), different letters indicate statistically significant differences (*p* < 0.05, one‐way ANOVA).
**Figure S3:** FgHMG1 triggers immune responses in *N. benthamiana* independent of enzymatic activity. (a) Representative *N. benthamiana* leaves exhibiting cell death symptoms at 5 days post‐inoculation (dpi) with Agrobacterium strains carrying indicated genes. (b) Immunoblot analysis of transient protein expression in *N. benthamiana* using an anti‐HA antibody (upper panel), with Ponceau S staining shown as the loading control (lower panel).
**Figure S4:** FgHMG1 overexpression does not alter filamentous growth in *F. graminearum*. (a) Relative *FgHMG1* transcript levels in overexpression strains relative to wild‐type PH‐1, quantified by RT‐qPCR with *FgACTIN* as internal reference. Different letters denote statistically significant differences (*p* < 0.05, one‐way ANOVA). (b) Colony morphology of PH‐1 and overexpression strains grown on PDA at 25°C for 3 days. Representative images are shown.
**Figure S5:** FgHMG1 protein enhances disease resistance in wheat against *F. graminearum*. Wheat seedling leaves were pretreated with purified FgHMG1 protein (from 100 nM to 2 μM) or the GFP protein (control) 24 h before *F. graminearum* inoculation. The values are presented as means ± standard deviation (*n* = 3).
**Figure S6:** FgHMG1 protein enhances disease resistance in rice against *Magnaporthe oryzae*. Rice seedlings leaves were pretreated with purified FgHMG1 protein or the GFP protein (control) 24 h before M. oryzae wild‐type Guy11 inoculation. Lesion numbers were counted within 1.5‐cm leaf segments. The values are the means ± standard deviation (*n* = 3). Significant differences (*p* < 0.05, one‐way ANOVA) are indicated by distinct letters.
**Figure S7:** FgHMG1 is highly conserved in the plant‐pathogenic fungi. The phylogenetic tree was created using RPB2 proteins sequences from 12 eukaryotes with MEGA X using the neighbour‐joining method (left). The purple shading indicates the level of homology with FgHMG1 (right).
**Figure S8:** FgHMG1‐induced resistance is stable across a range of temperatures. Wheat seedlings were pretreated with FgHMG1 protein that had been incubated at 20°C, 30°C, 40°C or 50°C, followed by *F. graminearum* PH‐1 inoculation. Lesion lengths were measured at 5 days post‐inoculation. Pretreatment with GFP protein was used as a control. Data represent mean ± standard deviation (SD) (*n* = 12). Box plots display median, interquartile range, min and max. Significant differences (*p* < 0.05, one‐way ANOVA) are indicated by distinct letters.
**Figure S9:** FgKMT6 is enriched at the FgHMG1 locus. ChIP‐qPCR assays revealed the enrichments of FgKMT6‐GFP at *FgHMG1* gene at 0–120 h post‐inoculation. ChIP‐ and input‐DNA samples were quantified by quantitative PCR assays. Data represent mean ± standard deviation from three biological replicates.

**Table S1:** Primers used in this study.

## Data Availability

The data that supports the findings of this study are available in the Supporting Information of this article.
