# Supplementary material for: H3K27me3‐Mediated Epigenetic Silencing of FgHMG1 Enables Fungal Host Immune Evasion
Source: Plant Biotechnol J. 2026 Jan 6;24(5):2843–57. doi: 10.1111/pbi.70530 (PMC13110184; doi:10.1111/pbi.70530)
Supplement: Supplementary file 1 — Figure S1: FgHMG1 induces cell death in N. benthamiana. Purified FgHMG1 protein (8 μM to 0.1 μM) or a GFP‐tagged control protein was infiltrated into N. benthamiana leaves. Representative leaf phenotypes photographed at 4 days post‐infiltration (dpi) (upper panel), corresponding leaves stained with trypan blue (lower panel). Figure S2: Xylanase activity of FgHMG1 and its site‐directed mutants. (a) Domain architecture of F. graminearum FgHMG1. (b) Expression and purification of GFP‐tagged proteins. (c) In vitro assays for the xylanase activity of indicated recombinant proteins. Xylanase activity of indicated proteins was determined using the 2,4‐dinitrosalicyclic acid (DNS) assay. The data are presented as means ± standard deviation (n = 3), different letters indicate statistically significant differences (p < 0.05, one‐way ANOVA). Figure S3: FgHMG1 triggers immune responses in N. benthamiana independent of enzymatic activity. (a) Representative N. benthamiana leaves exhibiting cell death symptoms at 5 days post‐inoculation (dpi) with Agrobacterium strains carrying indicated genes. (b) Immunoblot analysis of transient protein expression in N. benthamiana using an anti‐HA antibody (upper panel), with Ponceau S staining shown as the loading control (lower panel). Figure S4: FgHMG1 overexpression does not alter filamentous growth in F. graminearum. (a) Relative FgHMG1 transcript levels in overexpression strains relative to wild‐type PH‐1, quantified by RT‐qPCR with FgACTIN as internal reference. Different letters denote statistically significant differences (p < 0.05, one‐way ANOVA). (b) Colony morphology of PH‐1 and overexpression strains grown on PDA at 25°C for 3 days. Representative images are shown. Figure S5: FgHMG1 protein enhances disease resistance in wheat against F. graminearum. Wheat seedling leaves were pretreated with purified FgHMG1 protein (from 100 nM to 2 μM) or the GFP protein (control) 24 h before F. graminearum inoculation. The values are presented [file PBI-24-2843-s001.pdf]

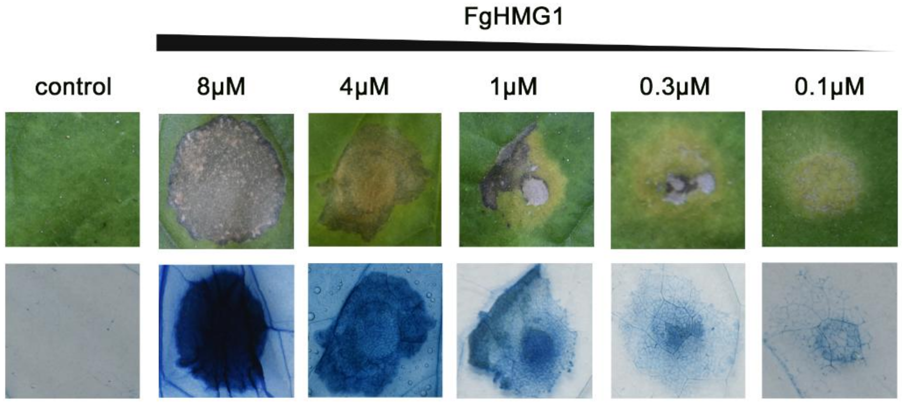

Figure S1. FgHMG1 induces cell death in *N. benthamiana.* Purified FgHMG1 protein (8 μM to 0.1 μM) or a GFP-tagged control protein was infiltrated into *N. benthamiana* leaves. Representative leaf phenotypes photographed at 4 days post-infiltration (dpi) (upper panel), corresponding leaves stained with trypan blue (lower panel).

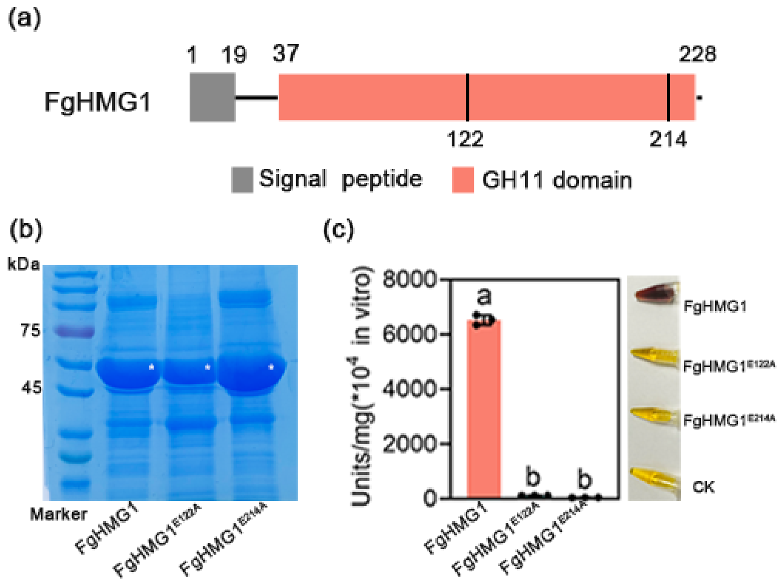

Figure S2. Xylanase activity of FgHMG1 and its site-directed mutants. (a) Domain architecture of *F. graminearum* FgHMG1. **(b)** Expression and purification of GFP-tagged proteins. **(c)** In vitro assays for the xylanase activity of indicated recombinant proteins. Xylanase activity of indicated proteins was determined using the 2,4-dinitrosalicyclic acid (DNS) assay. The data are presented as means ± standard deviation (n = 3), different letters indicate statistically significant differences ($p < 0.05$, one-way ANOVA).

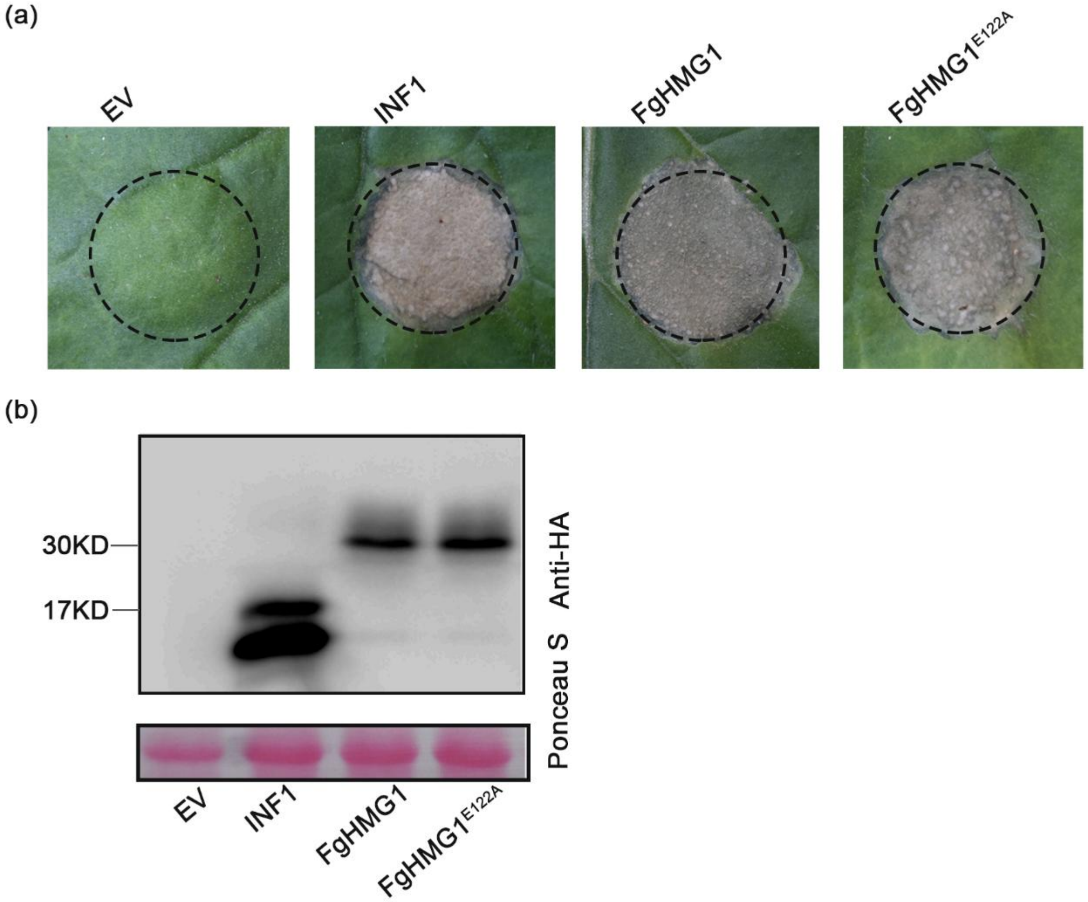

Figure S3. FgHMG1 triggers immune responses in *N. benthamiana* independent of enzymatic activity. (a) Representative *N. benthamiana* leaves exhibiting cell death symptoms at 5 days post-inoculation (dpi) with *Agrobacterium* strains carrying indicated genes. **(b)** Immunoblot analysis of transient protein expression in *N. benthamiana* using an anti-HA antibody (upper panel), with Ponceau S staining shown as the loading control (lower panel).

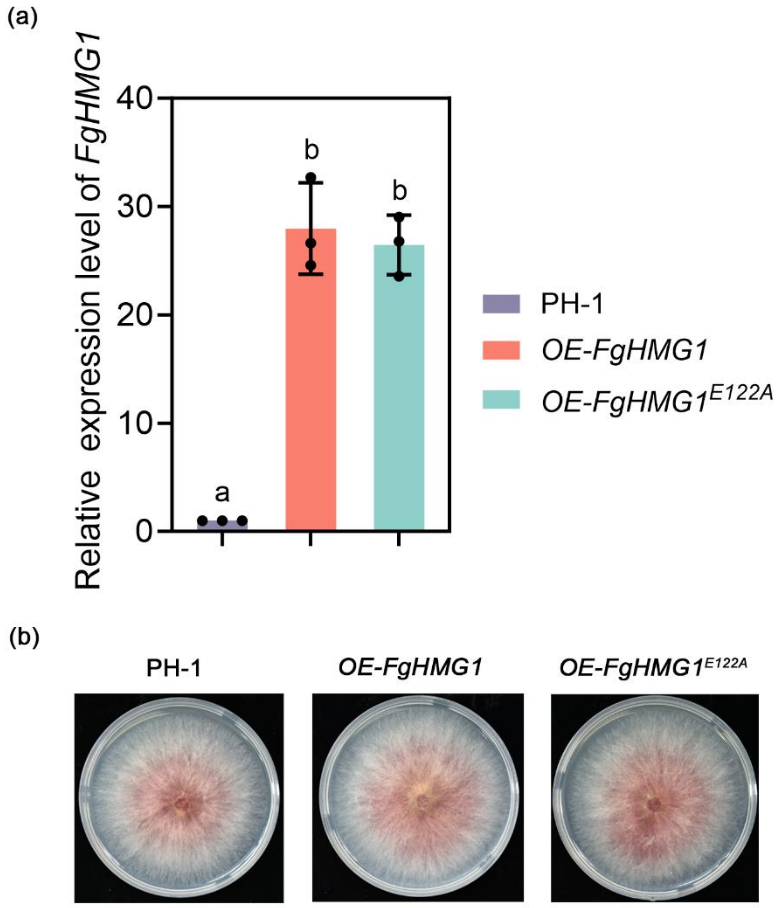

Figure S4. FgHMG1 overexpression does not alter filamentous growth in *F. graminearum.* **(a)** Relative *FgHMG1* transcript levels in overexpression strains relative to wild-type PH-1, quantified by RT-qPCR with *FgACTIN* as internal reference. Different letters denote statistically significant differences ($p < 0.05$, one-way ANOVA). **(b)** Colony morphology of PH-1 and overexpression strains grown on PDA at 25°C for 3 days. Representative images are shown.

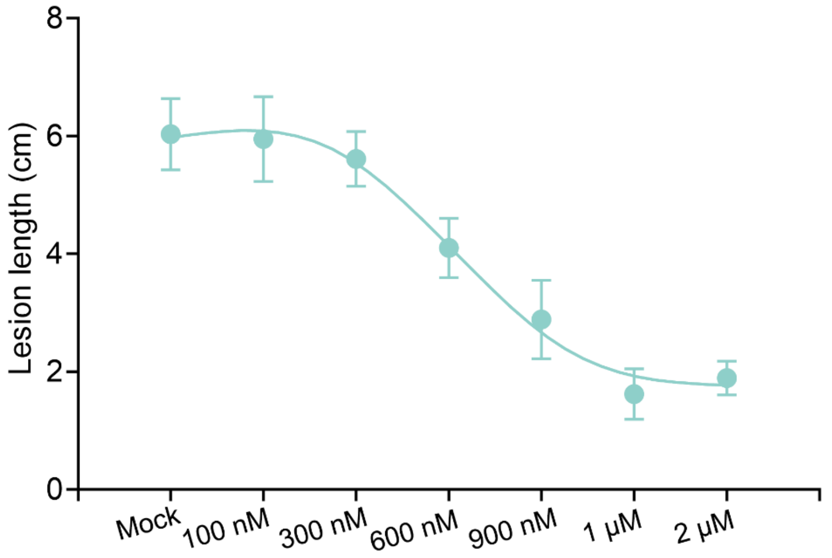

Figure S5. FgHMG1 protein enhances disease resistance in wheat against *F. graminearum*. Wheat seedling leaves were pretreated with purified FgHMG1 protein (from 100 nM to 2 μM) or the GFP protein (control) 24 hours before *F. graminearum* inoculation. The values are presented as means ± standard deviation (n=3).

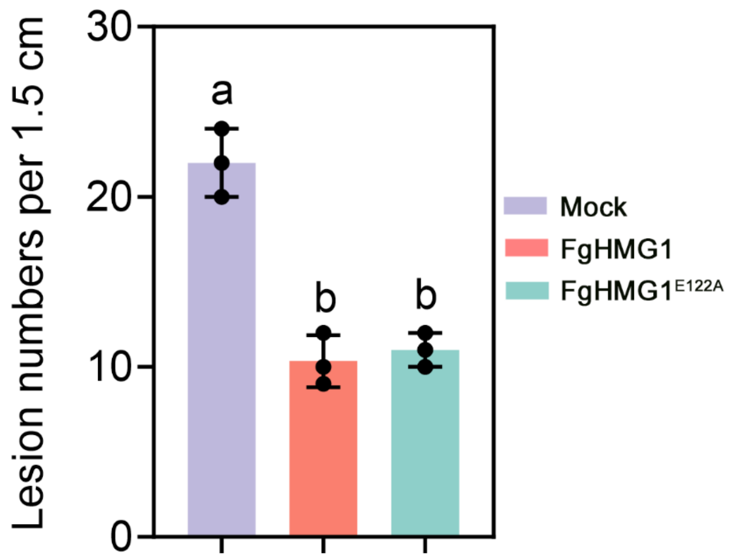

Figure S6. FgHMG1 protein enhances disease resistance in rice against *Magnaporthe oryzae*. Rice seedlings leaves were pretreated with purified FgHMG1 protein or the GFP protein (control) 24 hours before *M. oryzae* wild-type Guy11 inoculation. Lesion numbers were counted within 1.5-cm leaf segments. The values are the means ± standard deviation (n=3). Significant differences ($p < 0.05$, one-way ANOVA) are indicated by distinct letters.

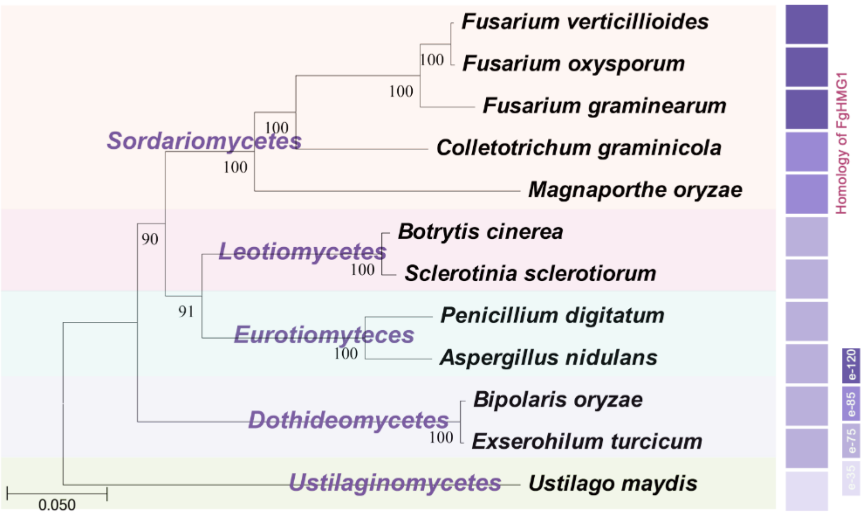

Figure S7. FgHMG1 is highly conserved in the plant-pathogenic fungi. The phylogenetic tree was created using RPB2 proteins sequences from 12 eukaryotes with MEGA X using the neighbor-joining method (left). The purple shading indicates the level of homology with FgHMG1 (right).

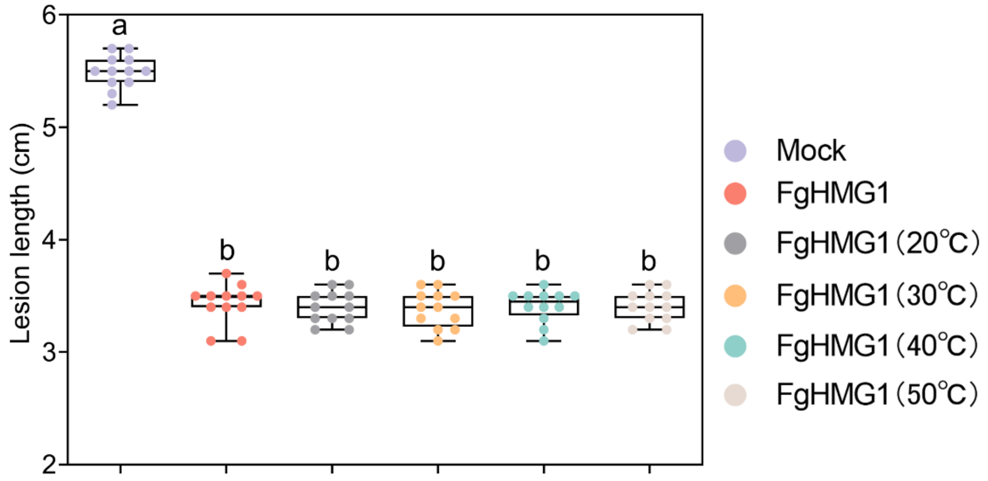

Figure S8. FgHMG1-induced resistance is stable across a range of temperatures. Wheat seedlings were pretreated with FgHMG1 protein that had been incubated at 20, 30, 40, or 50°C, followed by *F. graminearum* PH-1 inoculation. Lesion lengths were measured at 5 days post-inoculation. Pretreatment with GFP protein was used as a control. Data represent mean ± standard deviation (SD) (n=12). Box plots display median, interquartile range, min, and max. Significant differences ($p < 0.05$, one-way ANOVA) are indicated by distinct letters.

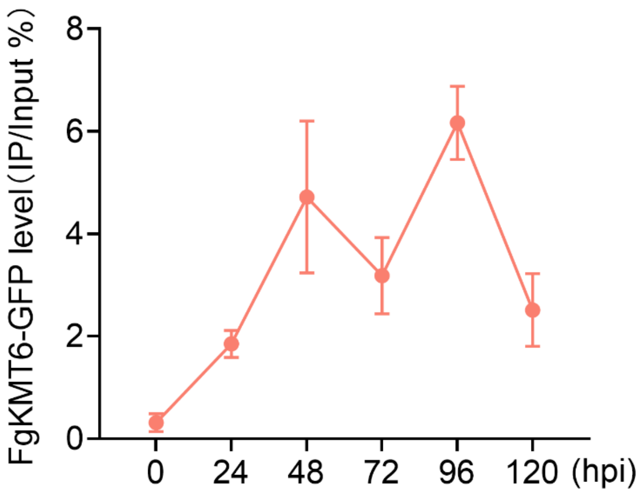

Figure S9. FgKMT6 is enriched at the *FgHMG1* locus. ChIP-qPCR assays revealed the enrichments of FgKMT6-GFP at *FgHMG1* gene at 0-120 hours post-inoculation. ChIP- and input-DNA samples were quantified by quantitative PCR assays. Data represent mean ± standard deviation from three biological replicates.
