# Supplementary material for: H3K27me3‐Mediated Epigenetic Silencing of FgHMG1 Enables Fungal Host Immune Evasion
Source: Plant Biotechnol J. 2026 Jan 6;24(5):2843–57. doi: 10.1111/pbi.70530 (PMC13110184; doi:10.1111/pbi.70530)
Supplement: Supplementary file 2 — Table S1: Primers used in this study. [file PBI-24-2843-s002.pdf]

Table S1. Primers used in this study.

Primer name	Sequence (5’→3’)
FgHMG1-PVX- F	TCCCGGGATGGTCTCCTTCACCTACCTTCT
FgHMG1-PVX-R	AAGGAAAAAAGCGGCCGCTCCAGAGACAGTCATGGTAGCCT
FgHMG2-PVX- F	TCCCGGGATGAAGCTGTCTGCTATTGC
FgHMG2-PVX- R	AAGGAAAAAAGCGGCCGCGTGGGATGGCTTGTAGGTAT
FgHMG3-PVX- F	TCCCGGGATGGCACTTGAAGACCATGG
FgHMG3-PVX- R	AAGGAAAAAAGCGGCCGCCTCGTTGTCCTTCTCCTCCT
FgHMG4-PVX- F	TCCCGGGATGTTGTTTCAAACCACCGC
FgHMG4-PVX- R	AAGGAAAAAAGCGGCCGCTTTAGACTTGACAGGCTTGT
FgHMG5-PVX- F	TCCCGGGATGAAGTTCACTACCCTCGC
FgHMG5-PVX- R	AAGGAAAAAAGCGGCCGCGGCACGTTGACATTGAGCAA
FgHMG1^{E122A}-PVX-F	TACTACATCGTCGAGAACTTCG
FgHMG1^{E122A}-PVX-R	CGAAGTTCTCGACGATGTAGTACGCAACGAGAGGGTTGC
FgHMG1^{E214A}-PVX-F	TCCCGGGATGGTCTCCTTCACCTACCTTCT
FgHMG1^{E214A}-PVX-R	AAGGAAAAAAGCGGCCGCTCCAGAGACAGTCATGGTAGCCTGGCCAGAGCTCTGGTAACCCGC
FgHMG1$^{\Delta SP}$-PVX-F	TCCCCCGGGGCTCCCAACCCTACCAAG
FgHMG1$^{\Delta SP}$-PVX-R	AAGGAAAAAAGCGGCCGCTCCAGAGACAGTCATGGTAGCCT
FgHMG1$^{\Delta SP}$-PR1-PVX-F	TCCCCCGGGATGGGATTTGTTCTCTTTTCGCA
FgHMG1$^{\Delta SP}$-PR1-PVX-R	CTTGGTAGGGTTGGGAGCTGACCTGGCACGGCAAGAGT
FgHMG1$^{\Delta SP}$-PR1-PVX-F2	GCTCCCAACCCTACCAAG
PVX-ID-F	TTACAGAGACGGCACCAAAG
PVX-ID-R	TTGCTGAGGTCCTCATTTGT
FgHMG1-PBinGFP-F	TCCCCCGGGATGGTCTCCTTCACCTACCTTCT
FgHMG1-PBinGFP-R	TGCTCTAGATCCAGAGACAGTCATGGTAGCCT

Pet28a-GFP-FgHMG1-F	GACGAGCTGTACAAGGGATCCGCTCCCAACCCTACCAAG
Pet28a-GFP-FgHMG1-R	TGGTGGTGCTCGAGTGCGGCCGCTCCAGAGACAGTCATGGTAGC
HPH-F	GGAGGTCAACACATCAATGC
HPH-R	CTACTCTATTCCTTTGCCCTCGGACGAG
FgHMG1-P1-F	CCGGCTGGTCTACTGTAT
FgHMG1-P1-R	ATCGTTGCGACCCTACTT
FgHMG1-P2-F	AGTTCAGCATGAACTGGAAC
FgHMG1-P2-R	GGGATGGTTAGCAAAAGTTC
FgHMG1-P3-F	GTCTCCTTCACCTACCTTCT
FgHMG1-P3-R	TAGGGCTGGTGCGCTTCT
FgHMG1-P4-F	ATCATGAGCTCTCCGCAT
FgHMG1-P4-R	CGTCGCTTTATCCGGTCTAG
NbBAK1-RT-F	ATTGCTGGAGGAGTTGCTGCCGG
NbBAK1-RT-R	CCACGTACAGCAGTGGTAACAT
NbSOBIR1-RT-F	CTTAGAAAAACTCTCTTTAGC
NbSOBIR1-RT-R	TATGGATTGGAGTGACATTATG
NbEF1α-RT-F	GTATGCCTGGGTGCTTGAC
NbEF1α-RT-R	ACAGGGACAGTTCCAATACCA
NbAcre31-qRT-F	AATTCGGCCATCGTGATCTTGGTC
NbAcre31-qRT-R	GAGAAACTGGGATTGCCTGAAGGA
NbPti5-qRT-F	CCTCCAAGTTTGAGCTCGGATAGT
NbPti5-qRT-R	CCAAGAAATTCTCCATGCACTCTGTC
NbPR1-qRT-F	CCGCCTTCCCTCAACTCAAC
NbPR1-qRT-R	GCACAACCAAGACGTACTGAG
NbPR2-qRT-F	AGGTGTTTGCTATGGAATGC

NbPR2-qRT-R	TCTGTACCCACCATCTTGC
NbWRKY33-qRT-F	TGCAGCAGATACAAATTTCCATC
NbWRKY33-qRT-R	ACAGCTGCCAATCAATCTATAG
gpda-F	CACCTTCAGTGGACTCGAG
gpda-R	TGTGATGTCTGCTCAAGCGG
OE- FgHMG1-F	CCGCTTGAGCAGACATCACAATGGTCTCCTTCACC TACCT
OE- FgHMG1-R	CAAAATAGGCATTGATGTGTTGACCTCCTTATCCAG AGACAGTCATGGTAG
TaPR1-qRT-F	GAGAATGCAGACGCCCAAGC
TaPR1-qRT-R	CTGGAGCTTGCAGTCGTTGATC
TaPR5-qRT-F	CTTCTACATCAAGAACAACTG
TaPR5-qRT-R	CAGTCGCCGGTCTGGCAG
TaERF113-qRT-F	GAACAGAGGGAGACCTTG
TaERF113-qRT-R	TTCAATTTTGCAAACAATGT
TaGAPDH-qRT-F	TTAGACTTGCGAAGCCAGCA
TaGAPDH-qRT-R	AAATGCCCTTGAGGTTTCCC
FgHMG1-qRT-F	TTCCAGCAGTACTGGTCCGT
FgHMG1-qRT-R	CAGCAAGGATCTGGTAGTCG
FgACTIN-qRT-F	GAGGGTACGTCGCATCAT
FgACTIN-qRT-R	TGACGAGAGCAGCAACTT
